# Men’s health and co-residence with older generations in Russia: better or worse?

**DOI:** 10.1136/jech-2017-209896

**Published:** 2017-12-20

**Authors:** Natalia Vadimovna Permyakova, Sunnee Billingsley

**Affiliations:** 1 Department of Social Statistics and Demography, University of Southampton, Southampton, UK; 2 Department of Sociology, Stockholm University, Stockholm, Sweden

**Keywords:** self-rated health, Eastern Europe, housing, social and life-course epidemiology

## Abstract

**Background:**

Previous studies show contradictory findings on the relationship between health and intergenerational living arrangements (ILAs), which may be due to variation in who selects themselves into and out of ILA. Addressing the selectivity into ILA and the health of the older generation, we assess whether there is a health-protective or health-damaging effect of ILA. We locate our study in the Russian context, where ILA is prevalent and men’s health has become a public health issue.

**Methods:**

We apply a fixed-effects logistic regression to self-rated health status of 11 546 men aged 25 years or older who participated in at least two waves in the Russian Longitudinal Monitoring Survey from 1994 to 2015. To further isolate the health effect of ILA, we observe only associations after transitioning into or out of ILA.

**Results:**

A transition into co-residence with an unhealthy older generation increases men’s odds of reporting poor health (OR=0.64, CI 0.44 to 0.93). A transition out of co-residence with a healthy older generation decreases men’s odds of reporting fine health by 63% (OR=0.37, CI 0.28 to 0.50), whereas continuing to live with an unhealthy older generation decreases the odds by half (OR=0.49, CI 0.38 to 0.63).

**Conclusions:**

We reveal a health interlinkage between co-residing generations by finding a detrimental health effect of co-residence with an unhealthy older generation. No longer living with an older generation who was in fine health also negatively affects men’s health. Future studies should address heterogeneity related to the health of older generations, unobserved time-constant characteristics of younger generations and selectivity into/out of ILA.

## Introduction

Intergenerational living arrangements (ILAs) have the potential to meet diverse needs by pooling resources and maximising human, social, physical and economic capital, which, in turn, might affect the well-being of individuals. In addition, co-residential living arrangements, and living with an older generation in particular, can curb unhealthy behaviour through social control.^1–3^ However, sharing a living space with an older generation can also increase the risk of physical^2^ and mental^4 5^ health problems due to stress from informal caregiving and multiple roles.^6–11^ Research on whether co-residence with an older generation is positively or negatively related to health is inconclusive.^1–11^

Understanding the relationship between health and ILA is complicated by the possibility that those who select themselves into and out of ILA may be unique and those selection forces may vary across contexts. Income and health operate as important mechanisms for residential transitions^12^ and often motivate moving into or out of ILA.^13^ ILA would appear to negatively influence health if ill adults are more likely to live with parents, whereas a positive health association would appear if it is healthy adults who are more likely to offer ‘at-home’ informal care and support to other family members in need. Likewise, a negative health effect of ILA would be observed if an older generation exits an ILA due to poor health of the younger generation and a desire to relieve any burden of co-residence, whereas a positive health effect may appear if healthy adults are more likely to achieve financial independence and move out of ILA. To date, we know little about how ILA influences health if we account for confounding factors related to the transition into and out of ILA.

Relying on longitudinal data and the dynamic nature of ILA, this paper addresses the following questions: is living in an ILA positively or negatively related to health? Does this depend on whether the older generation is in poor health? Does the relationship persist or change once we account for transitions into/out of ILA? We provide insights into the conflicting findings on the protective and detrimental effect of ILA on health and propose a methodological approach that reduces possible bias from unobserved heterogeneity related to transitions into and out of ILA. This study is situated in Russia, where ILA may be an important public health issue. One-third of Russian families continue to reside in intergenerational households,^14^ which is two to three times a higher proportion than is usual in northern and western European countries.^15^ We study Russian men, who have unusually high mortality and poor health,^16^ and hope to learn more about living conditions that support improved health of this vulnerable population.

## Data and methods

### Data source

We use the Russian Longitudinal Monitoring Survey (RLMS), which is based on a multistage sampling design and is nationally representative. Our panel data span 1994 to 2015, excluding 1997 and 1999. Individuals in households are questioned about their socioeconomic position, health and living conditions. We include 11 546 men aged 25 years and older (78 123 observations) who participated in at least two waves. We excluded men younger than 25 years because the mean age of leaving the parental home is 24 years old in Russia^17^ and our focus is on adults who are expected to live independently from their parents.

### Variables

Our main outcome is a dichotomised version of a five-category self-rated health answer: (1) poor=very bad or bad health and (2) fine=average, good or very good health. This health measure is recognised as a universal predictor of mortality.^18^ We dichotomise our outcome due to small end points and the rejected assumption of an ordinal nature in the parallel assumption test. The chosen categorisation follows the convention in empirical research on mortality related to self-rated health.^19^

Our main independent variable is time varying and indicates both whether the respondent lives with a parent/grandparent/parent-in-law (henceforth ‘older generation’) or not and whether this older generation is in poor or fine health (a dichotomised self-rated health outcome reported individually by older generations themselves). To define whether men are living in ILA or not, we identify household relationships using the household roster, as reported by the head of the household. RLMS collects information annually on the household roster and defines a household as a group of people who live together and share income and expenditures, including unmarried dependants away for studies. The set of questions used to define the household roster is consistent across the waves and includes the date of birth, gender, number of months living in the household (or the reason of absence since the last survey) of each household member and their relation to each other (parent/child, grandparent/grandchild, parent-in-law/child-in-law, etc). After identifying men’s older generations in the household roster, we retrieve individual-level information on the older generation’s self-reported health status, asked as a question ‘How would you evaluate your health?’ We therefore have a four-category ILA variable: (1) not co-residing with an older generation or co-residing with an older generation who reported (2) poor, (3) fine or (4) missing health status. We are able to capture changes in both living arrangements and older generation’s health only at the time of each survey. In cases where more than one member of an older generation was co-residing, we categorised the older generation as having poor health if any co-residing older generation reported poor health status.

We adjust our findings for a set of time-varying covariates including age of the respondent (25–34, 35–44, 55–64, 65 and older), partnership status (living with a partner or not), educational level (incomplete secondary school (SS), complete SS, vocational SS or higher education) and economic activity (currently working or being on leave/not working).

### Analytical strategy

We apply a multivariate logistic regression for the binary outcome of men’s health status. For easier interpretation of the direction effects, we estimate odds of reporting fine health. We first observe the relationship between ILA and health using the most common past approach: we treat our longitudinal dataset as repeated cross-sectional data, adjusting SEs for non-independence of observations. This model addresses some unobserved heterogeneity because, unlike past research, our specification of ILA distinguishes between poor and fine health of a co-residing older generation.

In a second model, we apply a fixed-effects approach to remove unobserved heterogeneity related to men’s characteristics that are stable over time. Unlike linear regressions, a fixed-effects approach with logistic regression relies only on within-person variation in the response variable.^20^ This requirement results in a reduction of our sample to the 2808 men who experienced a change from fine to poor health or vice versa during the observation period.

Fixed-effects models, however, still leave bias from time-varying unobserved characteristics or a reversed causal relationship.^20^ As a further step in isolating a health effect of ILA, we observe health changes that were tied specifically to a change in ILA. In one subsample, we include only men who were not co-residing with an older generation in the first wave of their participation (2257 out of 2808 men). Using the fixed-effects approach, this selection strategy means that the health of a man who lives in an ILA is contrasted with his own health before he started to live in an ILA. By ensuring that we know health status before living with the older generation, we assess whether a change in x is strictly related to a change in y. In the second subsample, we include only men who were co-residing with an older generation in the first wave of their participation (551 out of 2808 men). Likewise, the health of a man who does not live in an ILA is contrasted with his own health before he stopped living in an ILA. This analytical strategy has been used to isolate causal processes in health and well-being research,^8 21–23^ but it has not yet been applied to studies on the health effect of ILA.

## Results

Online supplementary appendix A presents descriptive statistics. In the modelling approach that best approximates past research (table 1, model 1), we observe a statistically significant association between ILA and men’s health that confirms previous findings of both positive and negative relationships.^1 3^ The direction of the association between ILA and health of adult children depends on the health status of a co-residing older generation. Men’s health is positively associated with ILA when living with an older generation in fine health: their odds of reporting fine health increase by 47% (OR=1.47, CI 1.24 to 1.74) in comparison with men not living with an older generation. In contrast, living with an older generation in poor health is negatively associated with men’s health: they have 28% lower odds of reporting fine health (OR=0.72, CI 0.61 to 0.85).

10.1136/jech-2017-209896.supp1 Supplementary file 1

**Table 1:** Odds of being in fine health according to living arrangement with an older generation, Russian men, 1994–2015

	Model 1: cross-sectional		Model 2: fixed-effects	
	OR	95% CI	OR	95% CI
ILA				
Not living with an older generation	1		1	
Living with older generation in poor health	0.72	(0.61 to 0.85)	0.98	(0.80 to 1.20)
Living with older generation in fine health	1.47	(1.24 to 1.74)	1.97	(1.56 to 2.48)
Age groups				
25–34	1		1	
35–44	0.59	(0.51 to 0.68)	0.98	(0.80 to 1.21)
45–54	0.30	(0.26 to 0.35)	0.65	(0.50 to 0.83)
55–64	0.23	(0.20 to 0.27)	0.43	(0.32 to 0.57)
65+	0.14	(0.12 to 0.16)	0.19	(0.14 to 0.26)
Living with a partner				
Without partner	1		1	
With partner	1.25	(1.11 to 1.40)	1.90	(1.60 to 2.25)
Education				
Incomplete SS	1		1	
Complete SS	1.32	(1.19 to 1.48)	1.33	(1.15 to 1.54)
Vocational secondary education	1.38	(1.20 to 1.57)	1.51	(1.19 to 1.90)
Higher education	1.63	(1.42 to 1.87)	1.45	(1.02 to 2.05)
Economic activity				
Currently working	1		1	
Not working or on (un)paid leave	0.22	(0.20 to 0.24)	0.32	(0.29 to 0.36)
Person-years (n) and men (n)	78 123	11 546	25 038	2808

Source: authors’ own calculations based on the Russian Longitudinal Monitoring Survey, 1994–2015.

ILA, intergenerational living arrangement; SS, secondary school.

### Conditional fixed-effects analysis

In model 2 (table 1), we use fixed effects to account for unobserved heterogeneity and generate estimates only from the variation within a person’s observations. Unlike in a cross-sectional model, there is no association between living with an unhealthy older generation and a change in men’s health (OR=0.98, CI 0.80 to 1.20). The positive health association of living with an older generation in fine health persists in the fixed-effects model. Net of stable unobserved characteristics, men have almost double the odds of reporting fine health if they are living with a healthy older generation (OR=1.97, CI 1.56 to 2.48) in comparison with those living without an older generation. However, we still cannot state whether an ILA exerts a positive effect on men’s health or whether men who are healthier are the ones who are more likely to co-reside with an older generation in fine health.

### Transitioning into ILA and men’s health

Including only men who were not living in ILA in their first observation (2257 men), we observe whether the health status of an older generation matters for men’s health only for men who began this ILA during the period of observation. Fixed-effects logistic regression results in model 3 (table 2) show that the transition to living with an unhealthy older generation increases the odds of reporting poor health (OR=0.64, CI 0.44 to 0.93), but this is not the case when transitioning to an ILA with a healthy older generation (OR=1.25, CI 0.81 to 1.94). Model 3 therefore confirms a negative effect of ILA on men’s health when the older generation is in poor health and demonstrates that a positive health association may depend on factors influencing the transition into ILA.

**Table 2:** Odds of being in fine health according to living arrangement with an older generation, selected samples of Russian men by living arrangement at first observation, 1994–2015

	Model 3: fixed-effects, men NOT living in ILA at start			Model 4: fixed-effects, men living in ILA at start	
	OR	95% CI		OR	95% CI
Not living in ILA	1		Stopped living in ILA	0.37	(0.28 to 0.50)
Started living with older generation in poor health	0.64	(0.44 to 0.93)	Continued living with older generation in poor health	0.49	(0.38 to 0.63)
Started living with older generation in fine health	1.25	(0.81 to 1.94)	Continued living with older generation in fine health	1	
Person-years (n) and men (n)	20 112	2257		4926	551

ORs are adjusted for age, education, economic activity and living with a partner. Source: authors’ own calculations based on the Russian Longitudinal Monitoring Survey, 1994–2015.

ILA, i ntergenerational living arrangement.

### Transitioning out of ILA and men’s health

In the final specification, only men living with an older generation in their first moment of observation are included in the sample. In model 4, we observe the effect of exiting an ILA on men’s health and whether older generation’s health confounds the effect of a continued ILA. The reference category is now living with an older generation who reports fine health. Fixed-effects logistic regression results in model 4 (table 2) reveal that no longer living with an older generation in fine health decreases the odds of reporting fine health by 63% (OR=0.37, CI 0.28 to 0.50) and living with an older generation in poor health (relative to fine health) decreases the odds by half (OR=0.49, CI 0.38 to 0.63).

Results from models 3 and 4 correspond, although the positive effect of living with healthy older generations is not found once accounting for health before ILA (model 3). That health worsens when no longer living with an older generation in fine health is an interesting finding that may be because the ILA ended due to a sudden illness of the older generation (who had previously been in fine health), which resulted in hospitalisation/institutionalisation. We were not able to assess hospitalisation-related exits of an ILA.

### Sensitivity analyses

We conducted multiple sensitivity analyses to ensure that the estimates we observe are robust (presented in online supplementary appendix B). When including separate ILA variables for each type of older generation (parents, grandparents, parents-in-law), we find similar relationships between each ILA and our health outcome. Accounting for period effects, the number of adults in the household, the number of minors younger than 16 years old in the household, the death of an older generation as a source of exiting an ILA, men’s parental status (having at least one biological/adopted/stepchild of any age in the household) or partner’s health status also does not change the main findings. We find no mediating effect of household economic well-being (income quintiles and self-assessments of a financial well-being). The results are robust in a sample of men aged 45 years old or older and in a sample of men younger than 65 years old (men’s retirement age in Russia). Finally, our results are similar when including lagged self-rated health as an alternative approach to reducing health-related selection effects.

## Discussion

This paper reveals the interlinkage between the health of co-residing adult children and older generations. Our aim was to clarify the direction of the association between ILA and health and expand previous cross-sectional findings^1 3 9^ by adjusting the estimates for unobserved heterogeneity and selection into and out of ILA. A few studies used a fixed-effects approach when accounting for ILA and health,^8 23^ but they focused specifically on the effect of informal care on caregiver’s health and missed the potential selection effect of a co-residence with unhealthy parents.^10 22^ The association between ILA and health is likely biased through the selection of adult children into and out of ILA based on health. First, our findings confirm the relevance of ILA to men’s health and the existence of both negative and positive associations that are due to the older generation’s health status. Second, we reveal that the negative influence of co-residing with an unhealthy older generation exists even if we account for the influence of unobserved characteristics of men and their health before transitioning into or out of an ILA.

### Interpretation of the findings

Once accounting for the transition of men into and out of ILA, the fixed-effects model confirms a negative health effect of living with an unhealthy older generation. This particular form of ILA may entail a burden of informal caregiving or the pressure of financial provision. Indeed, stress from multiple family roles—providing financial and emotional support and sharing a living space with older kin who have poor health and possibly require informal care—has been linked to mental health problems and declining health of caregivers.^6–9 11^ Past research leads us to generally expect negative effects of caregiving on the health of women,^7^ but men are also involved in parental caregiving within a household when living together with a partner because they are more likely to act as a ‘caring team’.^24^ Health may additionally be negatively influenced by the psychological difficulty of observing a parent suffer from poor health, increased emotional support needs of a female caregiver or even a combination of those factors that can lead to the deterioration of relationships with a spouse and other family members.^7 25^

No longer living with an older generation who was in fine health also negatively affects men’s health. Because we do not know the circumstances related to the end of this ILA, the finding is open to interpretation. If an older generation suddenly fell into illness that necessitated professional care and institutionalisation, it may be that it reflects the strain associated with more seriously poor health. On the other hand, no longer having older kin so close could lead to diminished emotional support, which has been shown to be particularly important in the case of Russian ILA,^26 27^ and contribute to worsening health.^22^ Previous studies on living arrangements show that men’s health behaviour may be less positive and life expectancy may be lower when living alone.^28^ Unhealthy behaviour has been strongly linked to men’s high risk of cardiovascular diseases and premature mortality in Russia, especially heavy drinking.^29^ These explanations should be investigated in further research before drawing firm conclusions.

Our results suggest that one pathway to poor health for Russian men is co-residing with an unhealthy older generation. To prevent this deterioration of health, policies related to health, economic prosperity and ageing may be important to consider. Many factors may contribute to the high prevalence of ILA in Russia: a cultural preference toward intergenerational support,^26^ financial assistance in a context of relatively high unemployment rates^30^ and grandparental help with childcare.^31^ In addition, housing prices and mortgage interest rates have increased since the regime collapse in 1991,^32^ leaving younger generations of Russians with difficulty affording independent housing^30^ and having to rely on inheritance.^32^ Perhaps most pertinent, 95% of Russians aged 60–80 years old report having poor health^33^; older people in Russia are, therefore, likely to require help with daily activities, which they could get by moving into ILA or care homes. The choice to live in care homes or receive institutional care is restricted by the availability and affordability of such care in Russia and cultural norms to personally take care of family members.^26^ Low pensions and increased cost of living drive older individuals in Russia to continue working for income past the retirement age,^34^ which is an option limited by poor health and may lead to an increased likelihood of ILA. All of these factors may compound the stress of having an unhealthy older generation with the added obligation for the older generation’s well-being.

These pathways into ILA, which may be somewhat specific to contexts such as Russia, highlight the interdependency between generations. Multiple linkages between life-course trajectories in family systems are important to identify because they bring to light the opportunities and constraints that are shared by individuals given their similar social status and network.^35 36^ Taking this perspective, the effects of ILA are likely compounded by socioeconomic status (SES): Russian men with low SES are most likely to end up in ILA with an unhealthy older generation with low SES as well because SES is intergenerationally inherited^37^ and poor health is strongly linked to low SES.^38^ Our finding of an effect of ILA on health therefore suggests that there could be a double health penalty for Russian men with low SES.

### Limitations and directions for further research

Like many panel data sources, RLMS has non-random attrition (by gender, age, household structure, marital status and health conditions).^39^ We are less likely to capture Russian men in follow-up surveys than women because of poor health, high alcohol consumption and cardiovascular diseases, which are interrelated.^16^ However, we are more likely to observe men in an ILA due to the higher chance of response in a shared household than in one-person households. Confirming previous findings,^39^ additional analyses of our data show that self-rated health does not predict attrition in the next wave.

This study shows the importance of addressing heterogeneity in the health of older generations when studying the influence of ILA on adult’s health, as well as the role of selection effects related to entering or exiting an ILA. Exploring further heterogeneity in the degree of parental poor health and intensity of informal caregiving^10 11^ could further clarify the role of ILA, which is understudied in the Russian context of ageing population and poor health.^33^ Other sources of heterogeneity would be worth considering in conjunction with the linked lives of younger and older generations, such as the duration of ILA^4^ or quality of the relationships.^40^ Country-level moderators such as normative family context and the supply of employment, pensions, housing, formal care and care institutions should be explicitly considered as they may create a country-specific selection of adult children into and out of ILA^41^ and play a role in the risk of falling into poor health.

**What is already known on this subject**

Living in an intergenerational arrangement is relevant to men’s health, but whether it has a health-protective or health-damaging effect is still unclear.

**What this study adds**

The nature of the relationship between intergenerational living arrangements (ILAs) and men’s health depends on the health of the older generation: living with an older generation in poor health negatively influences men’s health, but no longer living with an older generation who was in fine health also negatively affects men’s health. Addressing the potential for selectivity in transitioning into and out of ILA is important to estimate how health is influenced by ILA.
