# Supplementary material for: Men’s health and co-residence with older generations in Russia: better or worse?
Source: J Epidemiol Community Health. 2017 Dec 20;72(3):179–84. doi: 10.1136/jech-2017-209896 (PMC5868530; doi:10.1136/jech-2017-209896)
Supplement: Supplementary file 1 [file jech-2017-209896supp001.pdf]

Article title: Men's health and co-residence with older generations in Russia: Better or worse?

Journal of Epidemiology and Community Health

Authors: Natalia Vadimovna Permyakova[1], Sunnee Billingsley[2]

[1] Department of Social Statistics and Demography, University of Southampton, University Road, SO17 1BJ Southampton, United Kingdom
[2] Department of Sociology, Stockholm University, S-106 91 Stockholm, Sweden
* Corresponding author: Natalia Vadimovna Permyakova (nvp1g13@soton.ac.uk; +44 7544 137 872)

Appendix A. Descriptive statistics for the total sample of person-waves of Russian men aged 25 years or older, 1994-2015

**Variables**	**Total sample**					
	**N, total**	**%**	**Having a change in self-rated health status**			
			**AND living without parents in the 1st observation**		**AND living with parents in the 1st observation**	
			**N**	**%**	**N**	**%**
**Self-rated health status**						
Poor	9,578	*12.3*	6,626	*33.0*	1,353	*27.5*
Fine	68,545	*87.7*	13,486	*67.1*	3,573	*72.5*
**Intergenerational living arrangements (ILA) status**						
Not living with an older generation	60,760	*77.8*	19,514	*97.0*	1,773	*36.0*
Living with older generation in POOR health	4,515	*5.8*	279	*1.4*	1,317	*26.7*
Living with older generation in FINE health	12,390	*15.9*	299	*1.5*	1,719	*34.9*
Living with older generation with missing health	458	*0.6*	20	*0.1*	117	*2.4*
**Age groups**						
25-34	20,071	*25.7*	1,069	*5.3*	1,085	*22.0*
35-44	18,477	*23.7*	2,409	*12.0*	1,360	*27.6*
45-54	16,095	*20.6*	4,265	*21.2*	1,250	*25.4*
55-64	12,254	*15.7*	5,199	*25.9*	775	*15.7*
65+	11,226	*14.4*	7,170	*35.7*	456	*9.3*
**Partnership status**						
Without partner	13,588	*17.4*	2,705	*13.5*	1,770	*35.9*
With partner	64,535	*82.6*	17,407	*86.6*	3,156	*64.1*
**Educational level**						
Incomplete SS	16,506	*21.1*	6,661	*33.1*	1,166	*23.7*
Complete SS	30,391	*38.9*	6,821	*33.9*	2,076	*42.1*
Vocational SE	14,816	*19.0*	3,440	*17.1*	888	*18.0*
Higher education	16,410	*21.0*	3,190	*15.9*	796	*16.2*
**Economic activity**						
Currently working	51,091	*65.4*	8,335	*41.4*	2,476	*50.3*
Not working or (un)paid leave	27,032	*34.6*	11,777	*58.6*	2,450	*49.7*
**Total N of observations**	78,123	*100.0*	20,112	*100.0*	4,926	*100.0*
**Total N of men**	11,546		2,257		551	

Source: own authors' calculations based on the Russian Longitudinal Monitoring Survey, 1994-2015

Appendix B. Sensitivity analysis results

Table B.1. Odds of being in fine health according to living arrangement with parent(s), grandparent(s) and parent(s)-in-law, total sample of Russian men, 1994-2015

Variables	Fixed-effects model	
	OR	CI 95%
ILA: parents		
Not living with parent(s)	1	
Living with parent(s) in POOR health	0.93	[0.70,1.22]
Living with parent(s) in FINE health	1.88	[1.39,2.54]
Living with parent(s) with missing health	1.12	[0.51,2.49]
ILA: grandparents		
Not living with grandparent(s)	1	
Living with grandparent(s) in POOR health	0.62	[0.22,1.76]
Living with grandparent(s) in FINE health	3.21	[0.77,13.27]
Living with grandparent(s) with missing health	0.95	[0.21,4.34]
ILA: parents-in-law		
Not living with parent(s)-in-law	1	
Living with parent(s)-in-law in POOR health	1.03	[0.77,1.39]
Living with parent(s)-in-law in FINE health	1.99	[1.40,2.85]
Living with parent(s)-in-law with missing health	1.80	[0.88,3.69]
Living with a partner		
Without partner	1	
With partner	1.89	[1.59,2.24]
Age groups		
25-34	1	
35-44	0.98	[0.80,1.21]
45-54	0.64	[0.50,0.83]
55-64	0.43	[0.32,0.56]
65+	0.19	[0.14,0.26]
Education		
Incomplete SS	1	
Complete SS	1.33	[1.15,1.53]
Vocational SE	1.51	[1.19,1.91]
Higher education	1.45	[1.02,2.05]
Economic activity		
Currently working	1	
Not working or on (un)paid leave	0.32	[0.29,0.36]
Observations	25038	

Note: 95% confidence intervals in brackets
Source: own authors' calculations based on the Russian Longitudinal Monitoring Survey, 1994-2015

Table B.2. Odds of being in fine health of 2,257 Russian men NOT living in ILA at start, fixed-effects logistic regression models, 1994-2015

Variables	(1)	(2)	(3)	(4)	(5)	(6)	(7)
ILA							
Not living in ILA	1	1	1	1	1	1	1
Started living with older generation in POOR health	0.65<br>[0.45,0.95]	0.67<br>[0.45,0.98]	0.64<br>[0.44,0.93]	0.64<br>[0.44,0.93]	0.65<br>[0.44,0.95]	0.65<br>[0.45,0.95]	0.66<br>[0.46,0.96]
Started living with older generation in FINE health	1.27<br>[0.81,1.97]	1.29<br>[0.83,2.02]	1.25<br>[0.81,1.94]	1.25<br>[0.80,1.94]	1.26<br>[0.81,1.97]	1.28<br>[0.83,1.99]	1.31<br>[0.85,2.04]
Started living with older generation with missing health	1.06<br>[0.30,3.79]	1.01<br>[0.29,3.49]	1.00<br>[0.29,3.43]	0.99<br>[0.29,3.43]	0.84<br>[0.24,2.95]	0.90<br>[0.26,3.07]	1.03<br>[0.30,3.58]
Living with a partner							
Without partner	1	1	1	1		1	1
With partner	1.46<br>[1.20,1.78]	2.28<br>[1.77,2.93]	2.08<br>[1.71,2.52]	2.09<br>[1.73,2.53]		2.09<br>[1.72,2.54]	2.01<br>[1.66,2.44]
Age groups							
25-34	1	1	1	1	1	1	1
35-44	1.85<br>[1.36,2.51]	0.87<br>[0.64,1.17]	0.83<br>[0.62,1.12]	0.83<br>[0.62,1.12]	0.83<br>[0.62,1.12]	0.84<br>[0.62,1.12]	0.91<br>[0.68,1.23]
45-54	3.07<br>[2.09,4.50]	0.63<br>[0.44,0.89]	0.57<br>[0.41,0.80]	0.57<br>[0.41,0.80]	0.59<br>[0.42,0.83]	0.57<br>[0.40,0.79]	0.67<br>[0.48,0.95]
55-64	4.64<br>[2.95,7.27]	0.42<br>[0.29,0.62]	0.39<br>[0.27,0.56]	0.38<br>[0.26,0.54]	0.41<br>[0.29,0.60]	0.37<br>[0.26,0.54]	0.48<br>[0.33,0.69]
65+	4.66<br>[2.76,7.87]	0.19<br>[0.12,0.28]	0.17<br>[0.11,0.25]	0.16<br>[0.11,0.24]	0.19<br>[0.13,0.28]	0.16<br>[0.11,0.24]	0.23<br>[0.15,0.34]
Education							
Incomplete SS	1	1	1	1	1	1	1
Complete SS	1.18<br>[1.00,1.39]	1.29<br>[1.10,1.51]	1.29<br>[1.10,1.52]	1.29<br>[1.10,1.52]	1.27<br>[1.08,1.50]	1.30<br>[1.10,1.52]	1.28<br>[1.09,1.51]
Vocational SE	1.25<br>[0.96,1.63]	1.36<br>[1.05,1.76]	1.37<br>[1.06,1.78]	1.37<br>[1.06,1.78]	1.36<br>[1.04,1.77]	1.36<br>[1.05,1.77]	1.35<br>[1.04,1.76]
Higher education	1.51<br>[1.01,2.27]	1.52<br>[1.02,2.25]	1.54<br>[1.04,2.28]	1.54<br>[1.04,2.28]	1.50<br>[1.00,2.23]	1.52<br>[1.02,2.26]	1.52<br>[1.02,2.26]
Economic activity							
Currently working	1	1	1	1	1	1	1
Not working or on (un)paid leave	0.40<br>[0.36,0.46]	0.32<br>[0.29,0.37]	0.32<br>[0.29,0.37]	0.32<br>[0.29,0.36]	0.32<br>[0.29,0.36]	0.34<br>[0.30,0.38]	0.34<br>[0.30,0.38]
Survey year	0.88						

	[0.87,0.89]					
Other adults aged 16+ in household						
No other adults		1				
+1 adult		0.85 [0.65,1.10]				
+2 adults		0.81 [0.61,1.07]				
+3> adults		0.88 [0.65,1.19]				
Minors aged 0-15 in household						
No minors		1				
1 minor		1.12 [0.97,1.28]				
2 or more minors		1.30 [1.06,1.60]				
Men's parental status						
No children in household			1			
Living with child(ren)			1.05 [0.92,1.21]			
Death of older generations between wave t and t-1						
No death occurred				1		
At least one parent/grandparent/parent-in-law died				0.96 [0.44,2.08]		
Partner's health						
Living without partner					1	
Living with partner in poor health					1.09 [0.89,1.34]	
Living with partner in good health					3.47 [2.84,4.25]	
Living with partner with missing health					2.45 [1.65,3.64]	
Household income quintile						
Quintile 1 (lowest)						1
Quintile 2						0.95 [0.84,1.07]
Quintile 3						0.99 [0.86,1.13]
Quintile 4						1.13

						[0.97,1.31]	
Quintile 5 (highest)						1.08<br>[0.92,1.27]	
Self-assessment of household's financial well-being status							
The poorest (scores 1-3)						1	
In the middle (scores 4-6)						1.28<br>[1.18,1.39]	
The richest (scores 7-9)						1.22<br>[0.95,1.57]	
Missing reports						0.83<br>[0.63,1.10]	
Lagged self-rated health							
Poor health							1
Fine health							1.19<br>[1.10,1.29]
Missing (1st wave)							1.54<br>[1.36,1.74]
Observations	20112	20112	20112	20112	20112	20112	20112

Note: 95% confidence intervals in brackets;
(1) – addition of period effects ('survey year');
(2) – addition of 'other adults aged 16+' and 'Minors aged 0-15' in household;
(3) – addition of men's parental status;
(4) – addition of the time-varying recorded deaths of older generations in household;
(5) – addition of partner's health status;
(6) – addition of household income quintiles and self-assessed financial well-being;
(7) – addition of the lagged variable of men's self-rated health (t-1).

Source: own authors' calculations based on the Russian Longitudinal Monitoring Survey, 1994-2015

Table B.3. Odds of being in fine health of 551 Russian men living in ILA at start, fixed-effects logistic regression models, 1994-2015

Variables	(1)	(2)	(3)	(4)	(5)	(6)	(7)
ILA							
Stopped living in ILA	0.49<br>[0.36,0.68]	0.41<br>[0.29,0.58]	0.37<br>[0.27,0.50]	0.38<br>[0.28,0.51]	0.39<br>[0.29,0.53]	0.37<br>[0.28,0.50]	0.37<br>[0.27,0.50]
Continued living with older generation in POOR health	0.50<br>[0.39,0.65]	0.50<br>[0.39,0.65]	0.49<br>[0.38,0.63]	0.49<br>[0.38,0.63]	0.49<br>[0.38,0.63]	0.49<br>[0.38,0.63]	0.49<br>[0.38,0.63]
Continued living with older generation in FINE health	1	1	1	1	1	1	1
Continued living with older generation with missing health	0.83<br>[0.45,1.52]	0.82<br>[0.44,1.52]	0.75<br>[0.41,1.37]	0.75<br>[0.41,1.37]	0.74<br>[0.40,1.35]	0.74<br>[0.40,1.36]	0.75<br>[0.41,1.37]
Living with a partner							
Without partner	1	1	1	1		1	1
With partner	1.21<br>[0.81,1.79]	1.20<br>[0.79,1.82]	1.28<br>[0.84,1.94]	1.25<br>[0.85,1.84]		1.24<br>[0.84,1.83]	1.25<br>[0.85,1.85]
Age groups							
25-34	1	1	1	1	1	1	1
35-44	2.45<br>[1.70,3.53]	1.28<br>[0.95,1.74]	1.30<br>[0.96,1.75]	1.29<br>[0.96,1.75]	1.28<br>[0.95,1.73]	1.31<br>[0.97,1.77]	1.28<br>[0.94,1.74]
45-54	3.21<br>[1.80,5.73]	0.85<br>[0.56,1.29]	0.87<br>[0.58,1.31]	0.87<br>[0.58,1.31]	0.88<br>[0.59,1.33]	0.87<br>[0.58,1.31]	0.86<br>[0.57,1.30]
55-64	4.28<br>[1.90,9.66]	0.60<br>[0.34,1.05]	0.62<br>[0.36,1.07]	0.61<br>[0.36,1.06]	0.62<br>[0.36,1.07]	0.61<br>[0.35,1.06]	0.61<br>[0.35,1.06]
65+	4.83<br>[1.67,14.00]	0.37<br>[0.18,0.76]	0.38<br>[0.19,0.78]	0.38<br>[0.18,0.77]	0.39<br>[0.19,0.79]	0.37<br>[0.18,0.75]	0.38<br>[0.18,0.78]
Education							
Incomplete SS	1	1	1	1	1	1	1
Complete SS	1.42<br>[1.03,1.96]	1.48<br>[1.07,2.04]	1.47<br>[1.07,2.03]	1.47<br>[1.07,2.03]	1.46<br>[1.06,2.01]	1.43<br>[1.03,1.97]	1.47<br>[1.07,2.03]
Vocational SE	2.41<br>[1.39,4.18]	2.36<br>[1.37,4.09]	2.31<br>[1.34,3.99]	2.30<br>[1.34,3.98]	2.35<br>[1.35,4.07]	2.24<br>[1.30,3.87]	2.29<br>[1.33,3.96]
Higher education	1.21<br>[0.57,2.58]	1.22<br>[0.58,2.57]	1.22<br>[0.58,2.58]	1.22<br>[0.58,2.57]	1.26<br>[0.59,2.66]	1.17<br>[0.55,2.47]	1.22<br>[0.58,2.58]
Economic activity							
Currently working	1	1	1	1	1	1	1
Not working or on (un)paid leave	0.36<br>[0.29,0.45]	0.33<br>[0.27,0.42]	0.33<br>[0.27,0.42]	0.33<br>[0.27,0.42]	0.34<br>[0.27,0.42]	0.35<br>[0.28,0.43]	0.33<br>[0.27,0.42]
Survey year	0.90						

	[0.88,0.93]					
Other adults aged 16+ in household						
No other adults		1				
+1 adult		0.83 [0.51,1.36]				
+2 adults		0.86 [0.49,1.52]				
+3> adults		1.18 [0.63,2.21]				
Minors aged 0-15 in household						
No minors		1				
1 minor		0.96 [0.73,1.26]				
2 or more minors		1.10 [0.74,1.63]				
Men’s parental status						
No children in household			1			
Living with child(ren)			0.96 [0.68,1.37]			
Death of older generations between wave t and t-1						
No death occurred				1		
At least one parent/grandparent/parent-in-law died				0.91 [0.63,1.32]		
Partner’s health						
Living without partner					1	
Living with partner in poor health					0.55 [0.34,0.88]	
Living with partner in good health					1.43 [0.96,2.12]	
Living with partner with missing health					1.03 [0.43,2.45]	
Household income quintile						
Quintile 1 (lowest)						1
Quintile 2						1.18 [0.93,1.51]
Quintile 3						1.13 [0.87,1.48]
Quintile 4						1.19

						[0.88,1.62]	
Quintile 5 (highest)						1.18<br>[0.93,1.51]	
Self-assessment of household's financial well-being status							
The poorest (scores 1-3)						1	
In the middle (scores 4-6)						1.32<br>[1.11,1.58]	
The richest (scores 7-9)						1.27<br>[0.74,2.20]	
Missing reports						0.80<br>[0.47,1.37]	
Lagged self-rated health							
Poor health							1
Fine health							1.03<br>[0.86,1.23]
Missing (1st wave)							0.95<br>[0.73,1.24]
Observations	4926	4926	4926	4926	4926	4926	4926

Note: 95% confidence intervals in brackets;
(1) addition of period effects ('survey year');
(2) addition of 'other adults aged 16+' and 'Minors aged 0-15' in household;
(3) addition of men's parental status;
(4) addition of the time-varying recorded deaths of older generations in household;
(5) addition of partner's health status;
(6) addition of household income quintiles and self-assessed financial well-being;
(7) addition of the lagged variable of men's self-rated health (t-1).

Source: own authors' calculations based on the Russian Longitudinal Monitoring Survey, 1994-2015

Table B.4. Odds of being in fine health, fixed-effects logistic regression models based on three selected samples of Russian men, 1994-2015

Variables	Men aged 45 years old or older		Men aged 25-64 years old		Men with no temporary attrition	
	OR	CI 95%	OR	CI 95%	OR	CI 95%
ILA						
Not living with an older generation	1		1		1	
Living with older generation in POOR health	1.19	[0.92,1.55]	0.86	[0.69,1.07]	0.92	[0.71,1.19]
Living with older generation in FINE health	2.65	[1.81,3.88]	1.80	[1.41,2.29]	1.87	[1.39,2.51]
Living with older generation with missing health	1.72	[0.92,3.22]	1.44	[0.77,2.72]	3.17	[1.49,6.71]
Living with a partner						
Without partner	1		1		1	
With partner	1.94	[1.59,2.37]	1.63	[1.29,2.06]	2.48	[1.99,3.11]
Age groups						
25-34	-	-	1		1	
35-44	-	-	0.98	[0.79,1.20]	0.96	[0.72,1.27]
45-54	1		0.64	[0.50,0.82]	0.62	[0.45,0.87]
55-64	0.68	[0.59,0.78]	0.40	[0.30,0.53]	0.48	[0.33,0.69]
65+	0.31	[0.25,0.38]	-	-	0.21	[0.14,0.32]
Education						
Incomplete SS	1		1		1	
Complete SS	1.31	[1.11,1.55]	1.21	[1.02,1.44]	1.27	[1.06,1.51]
Vocational SE	1.53	[1.17,2.01]	1.32	[1.00,1.75]	1.46	[1.09,1.95]
Higher education	1.51	[1.00,2.29]	1.20	[0.78,1.85]	1.33	[0.87,2.03]
Economic activity						
Currently working	1		1		1	
Not working or on (un)paid leave	0.31	[0.27,0.35]	0.34	[0.30,0.38]	0.30	[0.26,0.34]
Observations	18405		16349		16877	

Note: 95% confidence intervals in brackets

Source: own authors' calculations based on the Russian Longitudinal Monitoring Survey, 1994-2015
